# ERF‐related craniosynostosis: The phenotypic and developmental profile of a new craniosynostosis syndrome

**DOI:** 10.1002/ajmg.a.61073

**Published:** 2019-02-13

**Authors:** Graeme E. Glass, Justine O'Hara, Natalie Canham, Deirdre Cilliers, David Dunaway, Aimee L. Fenwick, Noor‐Owase Jeelani, David Johnson, Tracy Lester, Helen Lord, Jenny E. V. Morton, Hiroshi Nishikawa, Peter Noons, Kemmy Schwiebert, Caroleen Shipster, Alison Taylor‐Beadling, Stephen R. F. Twigg, Pradeep Vasudevan, Steven A. Wall, Andrew O. M. Wilkie, Louise C. Wilson

**Affiliations:** ^1^ Department of Surgery Sidra Medicine Doha Qatar; ^2^ Division of Clinical Surgery Weill Cornell Medical College Doha Qatar; ^3^ Department of Craniofacial Surgery Great Ormond Street Hospital London United Kingdom; ^4^ North West Thames Regional Genetics Service, Kennedy Galton Centre Northwick Park and St. Mark's Hospitals Harrow United Kingdom; ^5^ Clinical Genetics Service, Oxford Centre for Genomic Medicine Oxford University Hospitals NHS Foundation Trust, Nuffield Orthopedic Centre Oxford United Kingdom; ^6^ Clinical Genetics Group, MRC Weatherall Institute of Molecular Medicine University of Oxford Oxford United Kingdom; ^7^ Craniofacial Unit, Department of Plastic and Reconstructive Surgery Oxford University Hospitals NHS Trust, John Radcliffe Hospital Oxford United Kingdom; ^8^ Oxford Genetics Laboratories Oxford University Hospitals NHS Foundation Trust, The Churchill Hospital Oxford United Kingdom; ^9^ Department of Clinical Genetics West Midlands Regional Clinical Genetics Service and Birmingham Health Partners Birmingham United Kingdom; ^10^ Department of Clinical Genetics Birmingham Women's and Children's Hospitals, NHS Foundation Trust Birmingham United Kingdom; ^11^ Department of Craniofacial Surgery Birmingham Children's Hospital Birmingham United Kingdom; ^12^ Department of Clinical & Academic Ophthalmology Great Ormond Street Hospital London United Kingdom; ^13^ Molecular Genetics Laboratory, North East Thames Regional Genetics Service Great Ormond Street Hospital London United Kingdom; ^14^ Department of Clinical Genetics University Hospitals of Leicester, Glenfield Hospital Leicester United Kingdom; ^15^ Clinical Genetics Service Great Ormond Street Hospital London United Kingdom

**Keywords:** Chiari‐1 malformation, craniosynostosis, ERF, facial dysmorphism, intracranial pressure, phenotype

## Abstract

Mutations in the *ERF* gene, coding for ETS2 repressor factor, a member of the ETS family of transcription factors cause a recently recognized syndromic form of craniosynostosis (CRS4) with facial dysmorphism, Chiari‐1 malformation, speech and language delay, and learning difficulties and/or behavioral problems. The overall prevalence of *ERF* mutations in patients with syndromic craniosynostosis is around 2%, and 0.7% in clinically nonsyndromic craniosynostosis. Here, we present findings from 16 unrelated probands with ERF‐related craniosynostosis, with additional data from 20 family members sharing the mutations. Most of the probands exhibited multisutural (including pan‐) synostosis but a pattern involving the sagittal and lambdoid sutures (Mercedes‐Benz pattern) predominated. Importantly the craniosynostosis was often postnatal in onset, insidious and progressive with subtle effects on head morphology resulting in a median age at presentation of 42 months among the probands and, in some instances, permanent visual impairment due to unsuspected raised intracranial pressure (ICP). Facial dysmorphism (exhibited by all of the probands and many of the affected relatives) took the form of orbital hypertelorism, mild exorbitism and malar hypoplasia resembling Crouzon syndrome but, importantly, a Class I occlusal relationship. Speech delay, poor gross and/or fine motor control, hyperactivity and poor concentration were common. Cranial vault surgery for raised ICP and/or Chiari‐1 malformation was expected when multisutural synostosis was observed. Variable expressivity and nonpenetrance among genetically affected relatives was encountered. These observations form the most complete phenotypic and developmental profile of this recently identified craniosynostosis syndrome yet described and have important implications for surgical intervention and follow‐up.

## 1 INTRODUCTION

Prevalence estimates for craniosynostosis, defined as the premature fusion of one or more of the cranial vault sutures, have ranged from 3.1 to 6.4 per 10,000 livebirths (Cornelissen et al., 2016). Around 30% of patients with craniosynostosis are identified as syndromic, with associated phenotypic and neurodevelopmental anomalies or malformations, or a positive family history (Wilkie et al., 2010; Wilkie, Johnson, & Wall, 2017). Among those for which the molecular basis has been identified (Twigg & Wilkie, 2015), the commonest include Muenke, Crouzon, Pfeiffer, Apert, Saethre‐Chotzen and craniofrontonasal syndromes (Ko, 2016) and more recently TCF12‐related craniosynostosis (Goos et al., 2016; Sharma et al., 2013), but there are many other rarer monogenic and chromosomal causes (Lattanzi, Barba, Di Pietro, & Boyadjiev, 2017).

ERF‐related craniosynostosis was first described in 2013 in 12 unrelated families accounting for 7.1% of a cohort of 127 patients with undiagnosed clinically syndromic craniosynostosis, and 2.9% of a total cohort of 412 undiagnosed patients with syndromic or nonsyndromic craniosynostosis (Twigg et al., 2013). More recently, the overall prevalence in all syndromic craniosynostosis has been estimated at 2% and in clinically nonsyndromic craniosynostosis at 0.7% (Wilkie et al., 2017). It appeared to be associated particularly with sagittal and lambdoid synostosis, but also multisutural craniosynostosis and pansynostosis. Chiari‐1 malformations appeared to be more common, and there was a relatively high risk of pathologically raised intracranial pressure (ICP), behavioral problems, and speech and language delay. A presumptive diagnosis of Crouzon syndrome had been made for many of these patients. Examples of variable expression and nonpenetrance were also reported (Twigg et al., 2013).

Since the initial report, two patients with *ERF* mutations have been described in a cohort of 40 patients with sagittal or multisutural synostosis (Chaudhry et al., 2015) and three patients with *ERF* mutations have been described in a cohort of 309 individuals with craniosynostosis who did not have a prior molecular diagnosis (Lee et al., 2018). A recent exome sequencing study of 291 parent‐offspring trios with nonsyndromic midline craniosynostosis reported a novel frameshift *ERF* mutation in a father and his two offspring each of whom had nonsyndromic metopic synostosis (Timberlake et al., 2017). Elsewhere, a specific heterozygous ERF missense p.(Y89C) substitution has been found to cause Chitayat syndrome in four unrelated probands and one parent with hyperphalangism, characteristic facies, hallux valgus, and bronchomalacia (Balasubramanian et al., 2017). None was noted to have craniosynostosis although only one had been assessed by cranial computed tomography (CT), at 5.5 years of age.

Here, we report our experience of 16 unrelated probands and 20 additional family members with heterozygous *ERF* mutations confirming that they contribute significantly to the craniosynostosis caseload, and highlight particular issues of importance in the clinical management of patients and their wider families.

## 2 PATIENTS AND METHODS

### 2.1 Editorial policies and ethical considerations

Patients known to the U.K. supra‐regional craniofacial units at Great Ormond Street Hospital (London), the John Radcliffe Hospital (Oxford), and Birmingham Children's Hospital and who had been diagnosed since the initial description of ERF‐related craniosynostosis (Twigg et al., 2013) were included for analysis. Most results have been generated as part of our routine clinical assessment and diagnostic service. Additional patients were ascertained through the Genetics of Craniofacial Malformations study (approved by London Riverside Research Ethics Committee [REC], reference 09/H0706/20) and the Deciphering Developmental Disorders study (approved by Cambridge South REC, reference 10/H0305/83). All subjects consented to the acquisition of this dataset. None of the patients have been reported previously and none have been ascertained through family follow‐up of the initial cohort (Twigg et al., 2013).

Common to all three services, genetic investigation for patients with multisuture or suspected syndromic craniosynostosis and without a known familial etiology includes screening for mutations in *FGFR1* (Exon 7), *FGFR2* (Exons 8 and 10), *FGFR3* (Exons 7 and 10) and *TWIST1* (Exon 1) sequencing and multiplex ligation‐dependent probe amplification as a minimum. Those with normal results have further testing of *FGFR2* (Exons 3, 5, 11, 14–17), *EFNB1*, *ERF*, *TCF12*, *IL11RA* and in some instances array‐CGH chromosome testing (although exact protocols vary slightly between centers and clinicians).

Following a diagnosis of ERF‐related craniosynostosis, a family history was obtained for all probands. Parents were offered genetic counseling, testing for the mutation and, where indicated, mutation screening was offered for other “at‐risk” family members, in line with standard clinical genetics practice.

All probands and related children identified with a familial *ERF* mutation were evaluated through the craniofacial service. In the majority this included CT head scanning with three‐dimension reconstruction to visualize the cranial vault and, in some cases magnetic resonance imaging of the brain depending on departmental protocol. Those with confirmed craniosynostosis were evaluated by a multidisciplinary team drawn from plastic and maxillofacial surgery, neurosurgery, otolaryngology, dental surgery, developmental pediatrics, audiology, ophthalmology, speech and language therapy, psychology, and clinical genetics.

After detailed review of the medical history, including the identification of any potentially confounding variables, a clinical evaluation for craniofacial dysmorphology was completed. Other noncraniofacial phenotypic features were noted. Radiological review for Chiari‐1 malformation was undertaken in each case. Ophthalmological assessment included visual acuity, fundoscopy and, at one center, visual evoked potentials. Visual impairment was defined as worse than 0.3 LogMAR with refractive correction and both eyes open. Audiological assessment included a hearing test and otoscopy. Language assessments were selected from a battery of standardized tests used routinely in the United Kingdom and based on the child's age (Wiig, Secord, & Semel, 2006a, 2006b; Zimmerman, Pond, & Steiner, 2009). Speech was assessed using a nonstandardized assessment (Grunwell, 1995). Speech, expressive and receptive language were rated as being normal or with mild, moderate or severe delay determined by the test scores obtained. Similarly, age‐specific gross and fine motors skills were evaluated by developmental pediatricians with the severity of delay summarized as before. Developmental pediatricians and/or child psychologists evaluated learning and behavior. While the reports on these evaluative domains were complex and bespoke, for the purpose of this study the findings were stratified by severity of learning and behavioral difficulty. The systematic assessment concluded with a multidisciplinary debriefing and data were recorded prospectively on a standardized proforma. Developmental assessments were carried out on a regular basis for all probands as part of their clinical evaluation and follow‐up. Family members were assessed on an ad hoc basis and on the basis of their self‐reported clinical history.

## 3 RESULTS

A total of 16 apparently unrelated probands with suspected pathogenic *ERF* mutations were identified. Of the genetically‐related family members identified by family tree who consented for genetic testing, an additional 20 individuals with ERF mutations linked to those of the probands was found. The *ERF* mutations and associated phenotypes are summarized in Table 1. The case history for each individual is summarized in Supporting Information.

**Table 1:** ERF mutations and the associated phenotype

Patient (kindred)	Gender (age at last assessment—years)	Mutation and parental origin	Relationship to proband	Synostosis	Facial dysmorphism	Other phenotypic features	Chiari‐1 malformation
**1 (K1)**	**F (19)**	c.891_892delAG; p.(G299Rfs*9) de novo	**Proband**	**Bicoronal unilambdoid**	**OHT, exorbitism, malar hypoplasia,**	**Factor XI deficiency, celiac disease**	**Yes (early)**
**2 (K2)**	**M (11)**	**c.301C>T; p.(R101W) maternal (inferred)**	**Proband**	**Pansynostosis**	**OHT, exorbitism, malar hypoplasia**	**Asthma. Lactose intolerance.**	**Yes (early)**
3 (K2)	M (12)	c.301C>T; p.(R101W) maternal (inferred)	Brother	None	OHT	Unilateral polymicrogyria	No
4 (K2)	F (9)	c.301C>T; p.(R101W) maternal (inferred)	Maternal half‐sibling	None	None	None	No
**5 (K3)**	**F (7)**	**c.247C>T; p.(R83W) maternal**	**Proband**	**Pansynostosis**	**OHT, exorbitism, malar hypoplasia**	**Short, broad DP of thumbs, allergy to gelofusine**	**Yes (early)**
6 (K3)	M (6)	c.247C>T; p.(R83W) maternal	Brother	Sagittal bilambdoid	OHT, exorbitism, malar hypoplasia	Broad DP of thumbs, halluces	No
7 (K3)	F (adult)	c.247C>T; p.(R83W) maternal	Mother	*Suspected sagittal*	OHT, mild exorbitism	None	N/K
8 (K3)	M (5)	c.247C>T; p.(R83W) maternal	Maternal cousin	Sagittal, bilambdoid, unicoronal (squamosal)	None	None	No
9 (K3)	F (adult)	c.247C>T; p.(R83W) maternal	Maternal aunt	None	None	None	N/K
10 (K3)	F (adult)	c.247C>T; p.(R83W) unknown	Maternal grandmother	None	None	Ovarian carcinoma	N/K
11 (K3)	F (adult)	c.247C>T; p.(R83W) maternal	Maternal aunt	None	None	None	N/K
12 (K3)	M (adult)	c.247C>T; p.(R83W) maternal	Maternal uncle	None	None	ONHD	N/K
13 (K3)	F (6)	c.247C>T; p.(R83W) maternal	Maternal cousin	Unilambdoid unicoronal (squamosal)	OHT, mild exorbitism	None	No
14 (K3)	M (4)	c.247C>T; p.(R83W) maternal	Maternal cousin	Pansynostosis	OHT, mild exorbitism	None	No
**15 (K4)**	**M (10)**	c.1390_1391dupCC; p.(K465Lfs*67) paternal	**Proband**	**Pansynostosis**	**OHT, exorbitism, malar hypoplasia**	**Left proximal radio‐ulnar synostosis, C5/6 vertebral fusion, broad thumbs**	**No**
16 (K4)	M (adult)	c.1390_1391dupCC; p.(K465Lfs*67) unknown	Father	*Suspected sagittal*	OHT, exorbitism,	None	N/K
**17 (K5)**	**F (7)**	**c.248G>A; p.(R83Q) paternal**	**Proband**	**Pansynostosis**	**OHT, exorbitism, malar hypoplasia, medial epicanthic folds**	**Bilateral 4th toe clinodactyly**	**Yes (early)**
18 (K5)	M (adult)	c.248G>A; p.(R83Q) unknown	Father	Sagittal bicoronal	OHT, exorbitism, malar hypoplasia, medial epicanthic folds	Broad thumbs	Yes
**19 (K6)**	**M (7)**	c.1201_1202del; p.(K401Efs*10) unknown	**Proband**	**Pansynostosis**	**OHT, malar hypoplasia, frontal bossing, long philtrum, high arched palate, low‐set ears**	**Inverted nipples. Bilateral 5th finger clinodactyly, broad DP of halluces, sacral dimple**	**Yes**
**20 (K7)**	**M (19 mo)**	**c.202G>C; p.(G68R) paternal**	**Proband**	**Sagittal unilambdoid**	**Hydrocephalus, macrocephaly, dysplastic auricles**	**None**	**No**
21 (K7)	M (19 mo)	c.202G>C; p.(G68R) paternal	Dizygotic twin	Metopic	Dysplastic auricles	None	No
22 (K7)	M (adult)	c.202G>C; p.(G68R) unknown	Father	*Suspected sagittal*	None	None	N/K
**23 (K8)**	**M (1)**	**c.161A>G; p.(E54G) maternal**	**Proband**	**Unilambdoid**	**Medial epicanthic folds, short up‐turned nose**	**None**	**No but small posterior fossa**
24 (K8)	F (adult)	c.161A>G; p.(E54G) unknown	Mother of proband	*Suspected sagittal*	OHT, exorbitism	None	N/K
**25 (K9)**	**F (3)**	c.1201_1202delAA; p.(K401Efs*10) paternal	**Proband**	**Unicoronal**	**Long philtrum, short up‐turned nose,**	**Joint hypermobility**	**No**
26 (K9)	M (adult)	c.1201_1202delAA; p.(K401Efs*10) unknown	Father	None	OHT, mild malar hypoplasia and prognathism	None	N/K
**27 (K10)**	**F (5)**	c.547C>T; p.(R183*) unknown	**Proband**	**Sagittal**	**OHT, mild exorbitism, malar hypoplasia**	**None**	No
**28 (K11)**	**F (15)**	c.547C>T; p.(R183*) paternal	**Proband**	**Sagittal bilambdoid**	**OHT, mild exorbitism, malar hypoplasia**	**Short lateral metatarsals**	**No**
29 (K11)	M (17)	c.547C>T; p.(R183*) paternal	Brother	Sagittal bilambdoid	OHT, mild exorbitism, malar hypoplasia, low‐set ears	Short lateral metatarsals	No
30 (K11)	M (adult)	c.547C>T; p.(R183*) unknown	Father	None	OHT, mild exorbitism, malar hypoplasia	None	N/K
**31 (K12)**	**M (4)**	**c.157G>A; p.(G53R) unknown**	**Proband**	**Sagittal**	**OHT, exorbitism, malar hypoplasia, low‐set ears, open mouth appearance**	**None**	**No**
**32 (K13)**	**M (4)**	c.652C>T; p.(R218*) maternal	**Proband**	**Sagittal, bilambdoid (sphenoidal)**	**OHT, mild exorbitism**	**Oculocutaneous albinism familial short stature**	**No**
33 (K13)	F (adult)	c.652C>T; p.(R218*) unknown	Mother	None	OHT, mild exorbitism	None	N/K
**34 (K14)**	**M (9)**	**c.247C>T; p.(R83W) maternal**	**Proband**	**Sagittal bilambdoid**	**OHT, exorbitism**	**None**	**No**
**35 (K15)**	**F (15 mo)**	c.1270C>T; p.(Q424*) suspected de novo	**Proband**	**Sagittal bilambdoid**	**OHT**	**None**	**No**
**36 (K16)**	**M (14)**	**c.1A>T p.? suspected de novo**	**Proband**	**Pansynostosis**	**OHT, mild exorbitism, malar hypoplasia**	**Broad thumbs/first toes**	**Yes (early)**

*Abbreviations*: DP = distal phalanx; F = female; K = kindred; M = male; mo = months; N/K = not known; OHT = orbital hypertelorism; P = patient. *Note*. Unconfirmed clinical diagnoses are in italics. Where the parental origin has been stated it has been confirmed by direct mutation testing. Where parental samples were not available the origin has been recorded as “unknown”. For the parents of a proband who carried the same ERF mutation, the origin in them was generally “unknown” as the grand‐parental samples were not available. In K2 the parental origin could be inferred. In two patients the mutation was suspected to be de novo on clinical grounds but parental samples were not available. Mutation nomenclature is based on NM 006494 (cDNA) and NP 006485 (protein). The bold text represents the Probands.

### 3.1 Genotype

**Figure 1:**
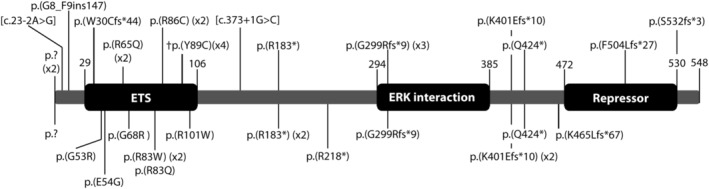
Domain structure of the ERF protein and the mutations identified. The mutations identified in the present cohort are shown underneath and the previously described mutations are shown above. †Refers to the heterozygous ERF missense substitution found to cause Chitayat syndrome (Balasubramanian et al., 2017)

Thirteen different heterozygous mutations (eight of which are reported for the first time) were identified in the 16 families comprising one mutation within the translation initiation codon, three nonsense mutations, three frame‐shifting mutations predicted to result in premature protein truncation, and six mutations predicted to result in missense substitutions (Figure 1).

Only one patient (P1) was confirmed by parental testing to have a de novo mutation. In two patients (P35, P36) the mutations are suspected to be de novo on clinical grounds but parental samples are awaited. In three families (K6, 10, 12) the parents were either unavailable or had declined testing. Of these, one parent was suspected to be affected in two families (one father because of exorbitism and one mother because of her facial appearance and history of mild learning difficulties) but have been classified as unknown for the purpose of this study. In one additional family (K2), although the parents were not available for assessment or testing, the available parental history and the identification of affected maternal half‐siblings infers maternal inheritance. In nine families one parent was found to carry the *ERF* mutation (five fathers; four mothers) but the grandparents and other relatives on that side had not been tested. In one further family where nine individuals have been found to carry the *ERF* mutation to date, it was traced back to the proband's maternal grandmother. Overall we observed 15 maternal transmissions (including three inferred) and seven paternal.

Three heterozygous ERF mutations (p.(R83W), p.(R183*), and p.(K401Efs*10)) were each shared by two families in our cohort. From our results we are not able to distinguish whether these mutations are recurrent or originate from a founder relative. Although the respective probands are not known to be related through available family histories, in each instance they originate from the same broad geographical area.

### 3.2 Craniosynostosis

All 16 probands and seven additional family members had radiological confirmation of craniosynostosis. A further four adults (P7, 16, 22, and 24) had suspected untreated (sagittal) synostosis.

**Figure 2:**
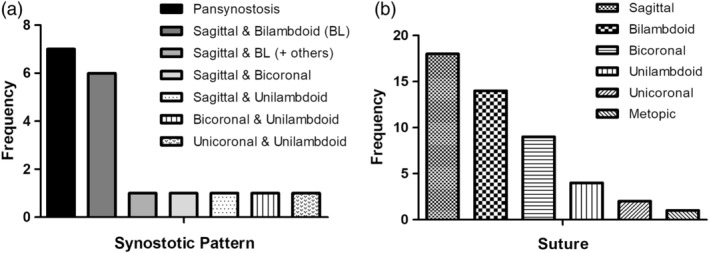
Craniosynostosis in the patient cohort. (a) The synostotic patterns identified among the 23 individuals evaluated radiologically. (b) The frequency of involvement of each suture or paired sutures

Seven patients exhibited pansynostosis, 11 exhibited multisuture synostosis and five exhibited single suture synostosis. Of the 11 patients with multisuture synostosis, seven included the sagittal and both lambdoid sutures. The synostotic patterns of the multisutural synostosis subgroup are shown in Figure 2a. The suture most frequently involved was the sagittal suture in 18 patients, followed by both lambdoid sutures in 14 patients. The frequency of individual and paired suture involvement is shown in Figure 2b.

**Figure 3:**
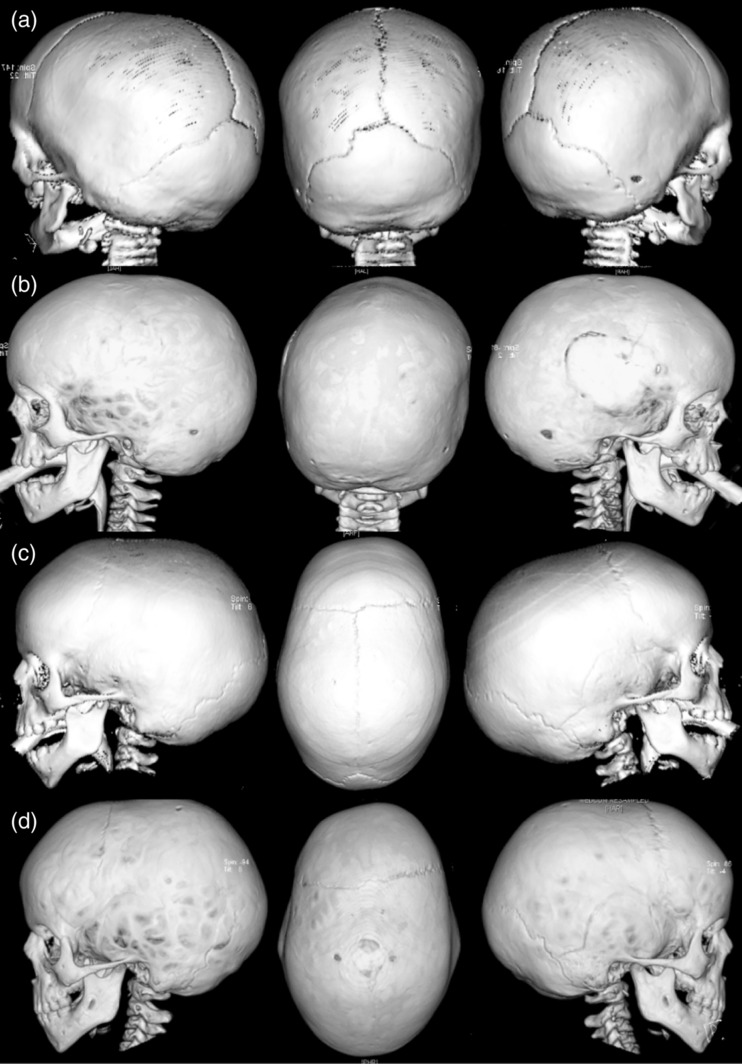
Selected 3D‐CT scan views from two probands illustrating the progressive nature of the craniosynostosis. (a) and (b) Patient 14 (K3): 3D‐CT images taken at age 0.8 and 2.7 years, respectively. At 0.8 years only the squamosal sutures were noted to be closed, progressing to pansynostosis with associated papilledema by 2.7 years. The site of the neurosurgical evacuation of a presumed spontaneous extradural bleed is also visible. Note the relatively normal skull shape. (c) and (d) Patient 8 (K3): 3D‐CT images taken at ages 1.9 and 4.7 years, respectively. At 1.9 years there was a scaphocephalic head shape with an indistinct sagittal suture suspicious of synostosis. By 4.7 years when clinical evidence of raised intracranial pressure became apparent, the craniosynostosis had progressed with clear involvement of the sagittal, superior bilambdoid, left inferior coronal, and left squamosal sutures

Importantly, only three of our 16 probands (P20, P25, and P35) had a sufficiently abnormal head shape and/or facial appearance to raise the suspicion of a craniofacial syndrome in the neonatal period. A further patient (P23) presented during infancy (Table 2). For the majority, the onset of craniosynostosis was insidious and progressive. This is illustrated by Patient 14 in whom early testing and monitoring was undertaken owing to his family history and in whom an evolving pattern of craniosynostosis was observed. In the neonatal period his head shape was normal and his fontanelle and sutures were patent clinically. CT scanning at 8 months of age showed bisquamosal synostosis and the inferior coronal sutures were felt to be indistinct radiologically but otherwise the sagittal, coronal and lambdoid sutures were patent (Figure 3a). He was kept under ophthalmological surveillance and had developed papilledema by 29 months of age when CT scans showed pansynostosis with a well preserved head shape (Figure 3b). Similarly, Patient 8 was first investigated at 23 months of age because of his family history. He had a mildly scaphocephalic head shape and his sagittal suture appeared indistinct on 3D‐CT suggestive of evolving sagittal synostosis (Figure 3c). By 4 years, 9 months of age when he developed blurred optic disc margins and raised ICP the craniosynostosis had progressed to involve both lambdoid sutures, the left coronal and squamosal sutures in addition to the sagittal (Figure 3d). Patient 32 had no evidence of craniosynostosis on skull X‐rays at 16 months when his hypertelorism and mild exorbitism raised the clinical suspicion of craniosynostosis, but he had developed sagittal, bilambdoid and fronto‐sphenoidal craniosynostosis by 39 months of age.

**Table 2:** Craniofacial and neurosurgical summary of the probands

Patient (kindred)	Age at presentation (months)	Raised ICP	Chiari‐1 malformation	VP shunt	1st cranial surgery (age in months)	Subsequent cranial surgery (age in months)
1 (K1)	42	Y	Y	N	PVE (48)	N
2 (K2)	49	Y	Y	N	PVE (51)	N
5 (K3)	52	Y	Y	Y	PVE (53)	N
15 (K4)	72	N	N	N	N	N
17 (K5)	42	N	Y	N	N	N
19 (K6)	28	Y	Y	Y	N	N
20 (K7)	<1	Y	N	Y	TCR (6)	N
23 (K8)	7	Y	N	N	PVE (8)	N
25 (K9)	<1	Y	Y	N	FOAR (13)	TCR (42)
27 (K10)	66	N	N	N	N	N
28 (K11)	180	N	N	N	N	N
31 (K12)	51	N	N	N	N	N
32 (K13)	30	N	N	N	N	N
34 (K14)	101	N	N	N	N	N
35 (K15)	<1	Y	N	N	PVE (8)	N
36 (K16)	24	Y	Y	N	PVE (28)	N

*Abbreviations*: FOAR = fronto‐orbital advancement remodeling; ICP = intracranial pressure; N = no; PVE = posterior vault expansion; TCR = total calvarial remodeling; VP = ventriculoperitoneal; Y = yes.

Two of the probands (P2, P5) had visual impairment from papilledema due to raised ICP at first presentation (49 and 52 months, respectively). Patient 5 has been left with permanent visual impairment. Her ophthalmology assessments showed bilateral optic disc atrophy with jerky horizontal and rotatory nystagmus and vision limited to hand movements on the right and light perception of the left. She uses Braille and requires one‐to‐one support at school. The most recent ophthalmology assessment for P2 showed mild disc pallor (worse on the left), a small angle left esotropia with latent nystagmus, and left amblyopia for which he has had patching. His vision has gradually improved achieving an acuity of 0.20 LogMAR in his better seeing eye.

A further seven probands had raised ICP at presentation while two family members (P8, P14) were observed to develop raised ICP as their craniosynostosis evolved. Chiari‐1 malformations were observed in seven of the 16 probands. In a further patient the cerebellar tonsils were reported to be low but had not reached the threshold for a Chiari‐1 malformation. All 10 probands with raised ICP and/or a Chiari‐1 malformation underwent cranial remodeling surgery +/− ventriculoperitoneal (VP) shunt placement and in each case the cranial surgery (usually in the form of posterior vault expansion) was performed within 12 months of presentation (Table 2). The siting of a VP shunt did not negate the need for cranial remodeling.

### 3.3 Facial dysmorphism

**Figure 4:**
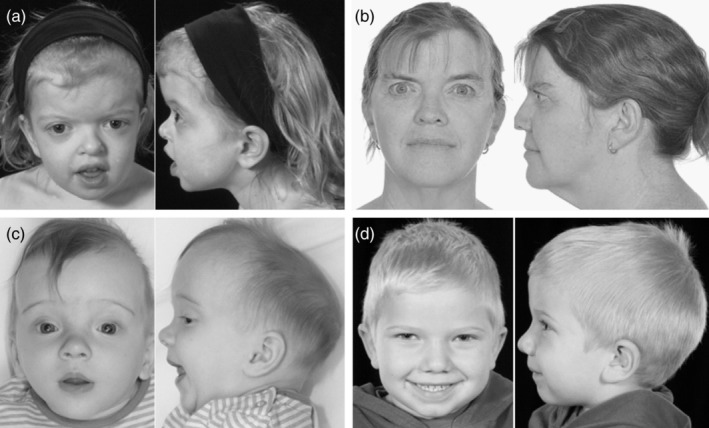
Spectrum of facial phenotypes in patients with ERF‐related craniosynostosis. (a) Patient 1 aged 3 years and (b) Patient 24 (adult) illustrating typical mild orbital hypertelorism and exorbitism with normal mid‐facial development. (c) Patient 35 aged 24 weeks illustrating scaphocephaly with a narrow occiput, mild orbital hypertelorism, and down‐slanting palpebral fissures with normal mid‐facial development. (d) Patient 8 aged 4 years illustrating a mildly elongated skull but normal facial appearance

Facial dysmorphism was present in 29 of the 36 subjects including all 16 probands. Orbital hypertelorism (OHT), with or without exorbitism and malar hypoplasia were the most common dysmorphic features (Figure 4). Despite this Crouzonoid triad, all but one patient exhibited a Class I occlusal relationship. Facial dysmorphism was not ubiquitous among this cohort as, notably, one family member who evolved a multisutural synostosis by 4 years, 8 months had a normal facial appearance (Figure 4). A summary of the phenotypic characteristics is shown in Table 1.

### 3.4 Developmental assessment

The developmental assessment profiles, as summarized in Table 3, show that most of the 16 probands demonstrated ophthalmological, audiological, neurological, speech and language, or behavioral anomalies. Ten of the 14 (71%) probands old enough to assess exhibited speech and/or receptive and expressive language delay, which generally responded well to therapy. In addition, seven of the family members reported speech delay and/or required speech and language therapy in childhood.

**Table 3:** Systems‐based summary of the multidisciplinary developmental assessment in subjects with *ERF* mutations

Patient (kindred)	Raised ICP	Audio‐visual development			Neurocognitive development					Other	Confounding variable
		Visual impairment	Otolaryngology	Hearing impairment	Speech and language delay	Gross motor delay	Poor fine motor skills/delay	Learning difficulties	Hyperactive +/or poor concentration		
**1 (K1)**	**Y**	**−**	**OME,**	**++ (SN)**	**++ (ex)**	**−**	**++**	**+**	**−**	**−**	**−**
			**Ear tubes**		**+ (re)**						
			**A&T**		**+ (Sp)**						
**2 (K2)**	**Y**	**++**	**OME**	**+ (co)**	**++ (ex)**	**+**	**−**	**+**	**+++**	**−**	**Fetal alcohol exposure**
					**++ (re)**						
					**+ (Sp)**						
3 (K2)	U/K	−	−	−	+ (ex)	−	−	+	+	Seizures	Fetal alcohol exposure
4 (K2)	U/K	−	−	−	−	−	−	+	+	−	Fetal alcohol exposure
**5 (K3)**	**Y**	**+++**	**OME, ear tubes, OSA, A&T**	**+ (co)**	**−**	**−**	**−**	**−**	**−**	**−**	**−**
6 (K3)	U/K	−	−	−	+ (ex)	−	−	−	+	−	−
					+ (re)						
					+ (Sp)						
7 (K3)	U/K	−	−	−	−	−	−	−	−	−	−
8 (K3)	U/K	– (P)	−	−	+ (ex)	−	−	−	−	−	−
					+ (re)						
					+ (Sp)						
9 (K3)	U/K	−	−	−	−	−	−	−	−	−	−
10 (K3)	U/K	−	−	−	−	−	−	−	−	−	−
11 (K3)	U/K	−	−	−	−	−	−	−	−	−	−
12 (K3)	U/K	−	−	−	−	−	−	+	−	−	−
13 (K3)	U/K	−	OME	−	+++ (ex)	+	−	+	+	−	−
					++ (re)						
14 (K3)	U/K	+ (P)	OME	+ (co)	++ (ex)	−	−	−	+	−	−
					++ (re)						
**15 (K4)**	**N**	**−**	**−**	**−**	**−**	**+**	**−**	**−**	**−**	**−**	**−**
16 (K4)	U/K	−	−	−	−	−	−	−	−	−	−
**17 (K5)**	**N**	**+**	**OSA**	**−**	**−**	**−**	**−**	**−**	**−**	**−**	**−**
			**A&T**								
18 (K5)	U/K	– (P)	−	−	−	−	−	−	−	−	−
**19 (K6)**	**Y**	**−**	**−**	**−**	**+ (ex)**	**+**	**−**	**−**	**−**	**−**	**Fetal alcohol exposure**
					**+ (re)**						
					**+ (Sp)**						
**20 (K7)**	**Y**	**−**	**OME**	**+ (co)**	**+ (ex)**	**−**	**−**	**−**	**−**	**−**	**−**
					**+ (re)**						
					**+ (Sp)**						
21 (K7)	U/K	−	−	−	+ (ex)	−	−	−	−	−	−
					+ (re)						
					+ (Sp)						
22 (K7)	U/K	−	−	−	−	−	−	+	−	−	−
**23 (K8)**	**Y**	**−**	**OME**	**−**	**−**	**−**	**−**	**−**	**−**	**−**	**−**
24 (K8)	U/K	−	−	−	−	−	−	−	−	−	−
**25 (K9)**	**Y**	**−**	**−**	**−**	**−**	**−**	**−**	**−**	**++**	**Autistic spectrum disorder**	−
26 (K9)	U/K	−	−	−	−	−	−	−	−	−	−
**27 (K10)**	**N**	**+**	**−**	**−**	**++ (ex)**	**+**	**+**	**++**	**−**	**Autistic spectrum disorder**	**16p13.11 microduplication**
					**++ (re)**						
					**+ (Sp)**						
**28 (K11)**	**N**	**−**	**−**	**−**	**+ (ex)**	**−**	**+**	**+**	**+**	**Seizures**	**−**
					**+ (re)**						
					**+ (Sp)**						
29 (K11)	U/K	−	OME	++ (co)	+ (ex)	−	−	+	+	−	−
					+ (re)						
					+ (Sp)						
30 (K11)	U/K	−	A&T	−	−	−	−	−	−	−	−
**31 (K12)**	**N**	**−**	**−**	**−**	**+ (ex)**	**+**	**+**	**+**	**−**	**−**	**Charcot–Marie‐tooth**
					**+ (re)**						
					**+ (Sp)**						
**32 (K13)**	**N**	**−**	**−**	**−**	**+ (ex)**	**+**	**−**	**+**	**+**	**−**	**Oculocutaneous albinism**
					**++ (re)**						
					**+ (Sp)**						
33 (K13)	U/K	−	−	−	+ (ex)	−	−	+	−	−	−
					+ (re)						
					+ (Sp)						
**34 (K14)**	**N**	**−**	**−**	**++ (SN)**	**+ (ex)**	**+**	**−**	**+**	**−**	**−**	**MYO15 sequence variants**
					**+ (re)**						
					**+ (Sp)**						
**35 (K15)**	**Y**	**−**	**OME**	**−**	**−**	**−**	**−**	**−**	**−**	**−**	**−**
**36 (K16)**	**Y**	**– (P)**	**Deviated septum/turbinate hypertrophy**	**−**	**+ (ex)**	**−**	**−**	**−**	**−**	**−**	**−**
					**+ (re)**						
					**+ (Sp)**						

*Abbreviations*: + = mild; ++ = moderate; +++ = severe; A&T = adenotonsillectomy; Co = conductive hearing loss; Ex = expressive language delay; ICP = intracranial pressure; OME = recurrent otitis media with effusions; OSA = obstructive sleep apnea; P = papilledema; Re = receptive language delay; SN = sensorineural hearing loss; Sp = speech delay; U/K = unknown.

The bold text represents the Probands.

Ten of the 16 (63%) probands exhibited poor gross motor and/or fine motor skills with deficits in gross motor control in five subjects, fine motor skills in two subjects and components of both in three subjects. Gross motor delay was a feature noted in the history of only one family member. Poor concentration and/or hyperactivity was observed in four of the 13 (31%) probands over 3 years of age and noted in the history of six of the 19 (32%) family members over 3 years of age. Six of the 12 (50%) probands and seven of the 19 (37%) family members older than 4 years needed support within their mainstream school or nursery. Importantly, of the nine probands with evidence of raised ICP, neurocognitive disturbance was identified in six and audio‐visual disturbance in four. However, of the seven probands without evidence of raised ICP, neurocognitive disturbance was identified in four and audio‐visual disturbance in three, suggesting that raised ICP was not the causative factor in these features.

Recurrent otitis media was identified in five (31%) probands and was a reported feature in the history of three family members. Associated hearing loss was variable.

## 4 DISCUSSION

We describe 36 previously unreported individuals from 16 kindreds in whom we have found 13 different heterozygous *ERF* mutations. Only one mutation in our cohort was confirmed to have arisen de novo, with a further two (P35, P36) suspected. The *ERF* mutation has been confirmed or can be inferred to have arisen from one of the parents in 10 of the probands. Two additional probands have one parent who is suspected to be mildly affected clinically.

Four of the *ERF* mutations found in our cohort have been reported previously (Twigg et al., 2013). One of those (p.(G299Rfs*9)) was confirmed in our patient to have arisen de novo and is therefore recurrent. For three others (p.R183*, p.K401Efs*10, and p.Q424*), we are unable to exclude the possibility of a founder effect since we have not been able to demonstrate a de novo origin and neither could Twigg et al. (2013) in their earlier cohort (Twigg et al., 2013). Mutations affecting the initiator codon have been reported twice previously (Chaudhry et al., 2015; Twigg et al., 2013) but the underlying nucleotide change in our patient was novel.

In keeping with the earlier findings, the predicted missense mutations in our cohort all occurred in highly conserved residues of the DNA‐binding ETS domain of the ERF protein between amino‐acids 29 and 106. The six mutations predicted to result in protein truncation were all located further towards the C‐terminus and to cause loss of the repressor domain, or ERK interaction and repressor domains, if they did not result in nonsense mediated mRNA decay. Overall the pattern of heterozygous mutations observed is consistent with a predominant haploinsufficiency mechanism of pathogenesis, as previously proposed (Twigg et al., 2013).

The most consistent clinical features of the probands include multisutural synostosis with the Crouzonoid triad of OHT, exorbitism and malar hypoplasia, as well as Chiari‐1 malformation, speech and language delay, poor fine and/or gross motor skills, and learning difficulties and/or hyperactivity, in keeping with previous findings (Twigg et al., 2013). While pansynostosis or sagittal and bilambdoid synostosis were the most frequent patterns of suture involvement accounting for 8 of 24 (33%) and 6 of 24 (25%), respectively, the sutural involvement in our cohort is more diverse than indicated from the initial report. Excluding the seven cases of pansynostosis, the sagittal suture was involved in 11 of 16 patients (69%) while both lambdoid sutures were involved in 7 of 16 (44%) and one lambdoid suture was involved in an additional 4 of 16 cases (25%). At least one coronal suture was involved in a third of cases (unilateral in three and bilateral in two). Given these findings we recommend a low threshold for testing for *ERF* mutations in patients with pansynostosis or multisuture synostosis of any pattern but particularly with sagittal and lambdoid involvement.

Importantly, ERF‐related craniosynostosis appears to present later than other craniosynostosis syndromes, with a median age at presentation of 42 months among the probands. Additionally, as a result of cascade screening we have been able to observe the evolution of the craniosynostosis in patients who may not otherwise have come to medical attention until later. Here, we have observed an indolent course of craniosynostotic development with progression to multisuture synostosis with raised ICP over the first few years. Hence, we believe that cascade screening and early testing of at‐risk infants is vital for the effective management of ERF‐related craniosynostosis including the avoidance of pressure‐related sequelae.

A notable feature in our cohort has been the relatively subtle change in head shape in many of the patients. We speculate that delayed evolution of the craniosynostosis in patients with *ERF* mutations may result in preservation of a normal head shape because it develops after the period of very rapid skull growth between the third trimester of pregnancy and the end of the first year of life. Moreover, while facial dysmorphism appears to be a common feature of ERF‐related craniosynostosis, we observed that it is usually symmetrically so. We speculate that the reason for this lies with the predominance of symmetrical synostotic patterns and this may contribute to delayed recognition of the condition.

The associated OHT and exorbitism is similar to that seen in Crouzon syndrome which was the commonest misdiagnosis in our series. It is interesting to speculate that the overlapping facial phenotypes result from a shared downstream constitutive activation of the RAS/MAPK pathway (Twigg & Wilkie, 2015). However, we have observed a number of distinctive differences between the two conditions, aside from the relative delay in presentation discussed above. Crucially, in the case of ERF‐related craniosynostosis mid‐facial hypoplasia was typically mild, and in no case was sufficiently severe to merit surgical intervention for airway management, ocular protection or appearance, even in adulthood. With the exception of one patient, all exhibited a Class I occlusal relationship. Moreover, the notably consistent pattern of developmental anomalies including speech and language delay, poor motor skills, and learning difficulties and/or behavioral problems typified by hyperactivity or poor concentration are not typical features of Crouzon syndrome. Encouragingly the speech and language and motor delays improved with supportive interventions. In addition, all the adult *ERF* mutation carriers were living independently as far as we could establish.

An important observation was that both neurocognitive and audio‐visual abnormalities were equally likely among the probands with raised ICP as those without. This would suggest that ICP alone is not solely responsible for these deficits but, rather, they are intrinsic features of the phenotype.

We note that the frequency of neurodevelopmental issues recorded in the adult *ERF* mutation carrying family members was much lower than expected given the results from the pediatric cohort. This may reflect a recall bias or alternatively, may suggest that the neurodevelopmental problems exhibit variable penetrance. Interestingly, four children from two kindreds within our cohort have been fostered or taken into social services care for neglect and in both families one biological parent carries the *ERF* mutation. We speculate that unrecognized learning and behavioral issues in unascertained adult *ERF* mutation carriers may have contributed to educational under‐achievement and/or social issues that may predispose to this occurrence.

Only two individuals in the entire cohort had sensory processing problems or features suggestive of autistic spectrum disorder, one of whom had a coincidental common recurrent 16p13.11 duplication which is a recognized neurosusceptibility variant enriched in patients with autism.

Overall, the observations in our cohort suggest that children with *ERF* mutations are likely to benefit from closer general pediatric surveillance and early interventions for their development and behavioral issues.

One patient had a radio‐ulnar synostosis, cervical vertebral fusions and broad thumbs and a further six patients were assessed as having broad thumbs and/or halluces. These orthopedic features overlap with those seen in other syndromic craniosynostoses, particularly FGFR2‐related Pfeiffer syndrome and may reflect overlapping downstream effector pathways.

None of our patient cohort had evidence of the hyperphalangy reported in Chitayat syndrome associated with short deviated index fingers and hallux valgus. All patients with Chitayat syndrome reported to date have had a specific heterozygous ERF p.Tyr89Cys missense substitution in the ETS domain, very close to mutations reported in ERF‐related craniosynostosis (Balasubramanian et al., 2017). Chitayat syndrome is also associated with facial dysmorphism (of a nature strikingly similar to that observed in our cohort), speech and language and motor delay (which is, again, similar in pattern to that observed in our cohort), and significant respiratory compromise from early childhood (which we did not observe in our cohort). Although none of the reported patients with Chitayat syndrome was considered to have craniosynostosis, only one had been assessed by cranial CT scan at 5.5 years of age. As our cohort demonstrates, the absence of a clearly abnormal skull shape in patients with ERF mutations does not exclude the possibility of craniosynostosis.

Somatic loss‐of‐function mutations in *ERF* have been reported in tumors including prostate, stomach and colorectal adenocarcinomas and Ewing's sarcoma (Bose et al., 2017; Huang et al., 2017) at frequencies of 3–5%. In several instances the somatic *ERF* mutations found in tumor tissue have been identical to the constitutional *ERF* mutations found in patients with ERF‐related craniosynostosis. There are other precedents for genes where identical mutations have been observed somatically in tumors and constitutionally in a variety of craniosynostosis and other dysmorphic syndromes, including genes encoding other components of the RAS‐MAPK pathway. As a general principle oncogenesis is a multistep process with progression dependent on the sequential accumulation of mutations within the tissue cells, such that the presence of a single constitutional mutation is not necessarily associated with a substantially increased cancer risk. We did not seek detailed cancer family histories in our cohort and have not undertaken extended testing to identify *ERF* carriers in the wider family of our cohorts and so we cannot address whether there is an increased cancer risk in these families.

In conclusion, ERF‐related craniosynostosis is a newly recognized disorder characterized by multisutural synostosis (with a predilection for pansynostosis or sagittal and bilambdoid involvement), facial dysmorphism with a mild Crouzonoid phenotype, Chiari‐1 malformation, delays in language development which generally resolve, behavioral abnormalities in the attention deficit and hyperactivity spectrum and mild learning disabilities which can usually be managed with support in mainstream education. The craniosynostosis may develop after birth in the first few years, evolve insidiously, and be associated with a relatively normal head shape. Cascade screening to identify children at risk in early childhood and close follow‐up of those identified as mutation carriers is strongly recommended to minimize the risk of serious visual sequelae of raised ICP and for early pediatric intervention for expressive and/or receptive language delay and behavioral issues. We advocate a low threshold for testing for *ERF* mutations in patients with multisutural or pansynostosis, or patients presenting with a Crouzonoid appearance and negative FGFR genetic screen. Crucially, *ERF* mutation carriers must be followed up regularly in the early years as the associated craniosynostosis is, unusually, indolent and progressive.

## Supporting information


**Appendix S1** A summary of the case histories of all 36 patients included in the study.
