# Supplementary material for: ERF‐related craniosynostosis: The phenotypic and developmental profile of a new craniosynostosis syndrome
Source: Am J Med Genet A. 2019 Feb 13;179(4):615–27. doi: 10.1002/ajmg.a.61073 (PMC6491982; doi:10.1002/ajmg.a.61073)
Supplement: Supplementary file 1 — Appendix S1 A summary of the case histories of all 36 patients included in the study. [file AJMG-179-615-s001.docx]

**Patient 1 (Kindred 1)**

**ERF c.891_892delAG; p.(G299Rfs*9)**

This 19 year old female is the second child of healthy unrelated parents with no relevant family history. An amniocentesis karyotype had been performed for increased risk of Down’s Syndrome and was normal. She delivered normally at term weighing 3.79 kg. She had recurrent febrile seizures which resolved. She presented to her local pediatric services with speech delay, particularly expressive language, at around 2 years, 6 months of age for which she had speech therapy. Her gross motor milestones were normal. She was referred to the craniofacial service at 3 years, 6 months of age with suspected Crouzon syndrome. She had a broad forehead, hypertelorism with down slanting palpebral fissures, exorbitism, and a high narrow palate but a class 1 incisor relationship with only mild maxillary hypoplasia. She was found to have bicoronal and lambdoidal synostosis with swollen optic discs, evidence of obstructive sleep apnoea and bilateral otitis media with effusions for which she had an adeno-tonsillectomy with grommets followed by a vault expansion aged 4 years. Her initial mild conductive hearing loss has progressed to bilateral moderate low frequency loss with a sensorineural component for which she has hearing aids. She has hypermetropia. A review of her communication skills at 10 years 11 months showed receptive and expressive language skills within the average range but she has needed some support within her mainstream school for mild learning difficulties. She also has a significant fine motor impairment. She has a Factor XI deficiency believed to be coincidental. Screening for Muenke syndrome, FGFR2 and TWIST mutations was normal and she was subsequently found to have a *de novo* heterozygous ERF c.891_892delAG; p.(G299Rfs*9) mutation.

**Patient 2 (Kindred 2)**

**ERF c.301C>T; p.(R101W)**

This 11 year old boy is the second of four children born to the same mother. He was delivered normally at 35 weeks gestation weighing 2.1kg with a head circumference of 32.5cm. At 6 months of age he was taken into care because of neglect and he has subsequently been adopted. When assessed at 7.5 months of age he was noted to have mild generalised delay with hypotonia, a wide anterior fontanelle, a large tongue and single palmar creases. His anterior fontanelle was still patent 1.5cm across at 1 year of age. By 2 years of age his hypotonia was resolving and his motor skills were within normal limits. He had moderate expressive language delay. At 3 years, 3 months of age he was referred for ophthalmological assessment because of a squint and a short history of clumsiness and tripping. He was found to have bilateral optic disc swelling. His OFC was 50cm (9-25^th^ centile) and there was no focal neurological deficit. MRI brain showed a partially empty sella and patulous optic nerve sheaths and benign intracranial hypertension was suspected. Lumbar puncture confirmed raised opening pressure which did not respond to acetazolamide or prednisolone. He was referred to the craniofacial team at 4 year, 1 month where he underwent a posterior vault expansion. A post-operative CT head scan showed evidence of multisuture synostosis. Subsequent multidisciplinary assessment found bilateral otitis media with effusions and mild conductive hearing loss, minimal left esotropia with no significant refractive error for which he had patching. He had hypertelorism with mild exorbitism, a wide mouth and a Class 1 jaw relationship. His limbs were normal. His receptive and expressive language skills had normalized but he was noted to have poor concentration. Behavioral screening at 11 years of age using a standardized strengths and difficulties parent questionnaire showed ‘close to average’ scores in all domains (Emotional stress; Behavioral difficulties; Difficulties getting along with other children; Kind and helpful behavior) except hyperactivity which was scored ‘very high’.

**Patient 3 (Kindred 2)**

This 12 year old boy is the older brother of patient 2. He was delivered normally at term. There was a suspected history of maternal drug and alcohol misuse. At 19 months of age he was taken into care because of neglect and malnutrition and he has subsequently been adopted. He had early speech delay but had caught up rapidly by 4 years of age. He attends mainstream school where he is felt to have normal learning ability but needs extra adult supervision because of difficulties with attention and concentration, impulsivity and difficulties interacting with peers. At 5 years of age he had a single afebrile seizure found to be associated with right frontotemporal EEG abnormalities and right perisylvian and parietal polymicrogyria on MRI brain scan. His structural brain abnormalities are suspected to be secondary to alcohol teratogenicity. His height and OFC were on the 75^th^ centile. He had hypertelorism and strongly resembled his brother facially. His gross and fine motor skills were age appropriate. His limbs were normal. Testing at 8 years of age showed that he carried the same ERF mutation as his brother and he was referred to the craniofacial service. He has not had cranial imaging but his ophthalmology assessments have been normal with no evidence of raised intracranial pressure.

**Patient 4 (Kindred 2)**

This 9 year old girl is the maternal half-sibling of patients 2 and 3. She has been fostered since birth and subsequently adopted. She was delivered normally at 38 weeks gestation. There were no neonatal problems. She began walking independently at 14 months of age and developed speech normally. She was referred because of her family history. On assessment at 5.5 years of age she had a height of 116.7cm (75-91^st^ centile) and head circumference of 52.2cm (50^th^ centile). Her head shape, facial appearance and other examination findings were normal, however, she was found to carry the same ERF mutation as her half-brothers. She attends mainstream school but, when last assessed at 7 years 2 months of age, was struggling particularly with numeracy and required additional support with her learning, concentration and behaviour. She had received a formal diagnosis of attention deficit hyperactivity disorder for which she was commencing medical treatment (Methylphenidate). There have been no problems with her vision or hearing and her general health is good.

**Patient 5 (Kindred 3)**

**ERF c.247C>T; p.(R83W)**

This girl is the third of five children born to healthy unrelated parents with no known history of craniosynostosis. Her pregnancy, delivery, birth weight and developmental milestones were all normal. She presented at 4 years, 4 months of age because she was bumping into things and was found to have reduced visual acuity and papilledema with a partial left VI nerve palsy. Transcranial pressure monitoring revealed elevated intracranial pressure. She underwent a ventriculo-peritoneal shunt with stabilization of her findings. A CT scan showed pansynostosis with severe copper beating and she was referred to the Craniofacial service. She was noted to have a normal head circumference on the 50-75^th^ centile, hypertelorism, mild exorbitism and an early class III dental occlusion pattern. She had short, broad distal phalanges of both thumbs. A presumptive diagnosis of Crouzon syndrome was made. In appearance, she resembled her mother. At 4 years, 5 months of age she underwent a posterior vault expansion. She subsequently developed bilateral optic atrophy with severe visual impairment limited to perception of light, colour and hand movements, and now uses Braille. She was found to have moderate obstructive sleep apnoea for which she had an adeno-tonsillectomy at 4 years, 11 months. She developed bilateral otitis media with conductive hearing loss for which she had bilateral grommet insertion age 7 years, 4 months. She attends a mainstream school with 1 to 1 help for her visual impairment. She is making normal academic progress and has no problems with speech and language, behaviour or dyspraxias.

Molecular genetic testing excluded Muenke syndrome and an extended FGFR2 mutation screen was normal as was TWIST mutation and deletion screening and a karyotype. She was subsequently found to have a heterozygous ERF c.247C>T; p.(R83W) mutation predicted to be pathogenic inherited from her mother.

**Patient 6 (Kindred 3)**

This boy was assessed at 3 years, 6 months of age along with his siblings because of the findings in their sister. His gestation, delivery, developmental milestones and general health had all been normal. He had a normal head circumference of 50.8cm (25^th^ centile) with a mildly scaphocephalic head shape, some frontal bossing, hypertelorism and mild exorbitism but a normal class 1 incisor relationship. Like his sister, he had broad distal phalanges of thumbs and great toes. He was found to have sagittal and bilambdoidal synostosis but there has been no evidence of raised intracranial pressure. His visual acuity, fundi and electrophysiology have been normal on repeat assessments. His hearing has been normal and there are no otolaryngological concerns. At his most recent assessment aged 6 years, 6 months, the only additional finding was significant behavioural problems assessed as resulting from a short attention span and easy distractibility.

**Patient 7 (Kindred 3)**

The mother of the proband was in good general health with no history of any significant medical or educational problems. She had a narrow head shape with hypertelorism, down slanting palpebral fissures, mild exorbitism and malar hypoplasia, high narrow palate and mild micrognathia. She had bilateral broad distal phalanges of thumbs but normal halluces. She has four siblings all of whom are felt to be in good general health.

**Patient 8 (Kindred 3)**

This 5 year old is one of two boys born to Patient 9 and her healthy unrelated partner. He was delivered by elective Caesarean section 5 days post-term after a normal pregnancy weighing 4.9kg. There were no neonatal problems. He was assessed at 22 months of age because of the family history. His motor milestones were normal but he was found to have mildly delayed receptive and expressive language skills and mild bilateral hypermetropia. At 22 months he had a height of 83cm, weight of 14.1kg and head circumference of 52.7cm. On examination he had scaphocephaly, a convexity over his closed anterior fontanelle, and blepharochalasis with small medial epicanthic folds but no significant hypertelorism or exorbitism. He had a Class 1 dental occlusion pattern. His CT scan demonstrated an indistinct sagittal suture but no other evidence of craniosynostosis. He was kept under ophthalmology and craniofacial surveillance. A speech assessment at 4 years, 4 months of age found that his receptive and expressive language skills were now age appropriate but he had a number of speech sound errors for which he was referred to speech & language pathology. On multidisciplinary craniofacial review at 4 years, 9 months he had blurred optic disc margins. He was admitted for combined intracranial pressure monitoring and sleep study which confirmed raised intracranial pressure unrelated to sleep. A repeat 3D-CT scan confirmed sagittal, bilambdoidal, left inferior coronal a squamosal synostosis. He underwent a posterior vault expansion with springs two weeks later. At his last assessment age 5 years he had made a good recovery from his surgery and his ophthalmology findings had improved. His development was within normal limits.

**Patient 9 (kindred 3)**

This lady is the maternal aunt of the proband and was assessed because of the family history. She had no history of any significant health or educational issues. She had been found to have normal vision with no significant ophthalmological abnormality on assessment at 29 years of age. On examination, she had a head circumference of 57.4cm with a tall narrow skull, but no significant scaphocephaly, and mild nasal deviation.

**Patient 10 (Kindred 3)**

This 59 year old lady has a history of ovarian cancer but no other history of note. On examination she had a head circumference of 59.4cm. She was found to carry the same ERF mutation as other affected family members.

**Patient 11 (Kindred 3)**

This lady is the maternal aunt of the proband and was assessed because of the family history. She had has a history of recurrent headaches but otherwise good health. She had a head circumference of 56cm with a normal appearance and skull shape and no obvious ridging. She was found to carry the same ERF mutation as other affected family members.

**Patient 12 (Kindred 3)**

This is a maternal uncle of the proband and was offered testing because of the family history. He had a learning difficulties at school. His vision was normal but ophthalmology assessment showed optic nerve head drusen (ONHD) on the temporal aspect of both discs resembling areas of blotchy pallor.

**Patient 13 (Kindred 3)**

This 6 year old girl is one of three children born to patient 11 and her healthy unrelated partner. She was assessed because of the family history of ERF mutation. She had a history of recurrent bilateral otitis media and some delay in her gross motor and language milestones but was otherwise in good health. She had a normal head shape with a metopic ridge, orbital hypertelorism and mild exorbitism. Her 3D CT scan showed multisutural synostosis involving the right lambdoid and left coronal and squamosal sutures. There was no ophthalmological evidence of raised intracranial pressure. She was confirmed to have delayed gross motor skills with moderate receptive language delay, moderate to severely delayed expressive language and disordered speech. Fine motor, problem solving and personal social skills were normal. She attends mainstream school with additional teaching support for reading. Her hearing was normal when last assessed at 6 years.

**Patient 14 (Kindred 3)**

This 4 year old boy was assessed at 8 months of age after he was investigated due to his family history and found to carry the familial ERF mutation. He was found to have mildly delayed receptive and expressive language and hearing impairment secondary to otitis media with effusions. His 3D CT scan showed bilateral squamosal synostosis only (**Figure 2A**). On later review at 23 months he was found to have moderately delayed expressive and receptive language skills and was referred to speech pathology. He had good social skills and age appropriate attention and listening skills. Shortly after that he presented acutely to emergency services with a presumed spontaneous extradural bleed from the right middle meningeal artery requiring neurosurgical evacuation. There was no evidence of trauma, non-accidental injury, metabolic or clotting disorders on wider investigation. At around 28 months of age, a routine ophthalmology review found reduced visual acuity and bilateral papilledema. Cranial CT scanning showed that he had progressed to a pansynostosis (**Figure 2B**). He underwent a posterior skull vault expansion with springs with complete resolution of his papilledema and visual acuity. His receptive and expressive language remained moderately delayed but had improved. He was noted to have reduced attention and concentration and to be easily distracted. His sleep study was normal. On his most recent review at 3 years 8 months he had a recurrent ear infection, but play audiometry indicated borderline hearing levels in the right ear and mild loss in the left ear. Unmasked bone conduction audiometry indicated normal underlying hearing in at least one ear. He had an intermittent divergent squint but normal acuities and optic discs. His communication skills remained borderline on the Ages & Stages questionnaire but all other developmental areas were on schedule. He had a reverse overjet of 1mm.

**Patient 15 (Kindred 4)**

**ERF c.1390_1391dupCC p.(K465Lfs*67)**

This 10 year old boy was born to unrelated Caucasian parents at 37 weeks gestation by breech presentation weighing 3.32kg. He did not walk until 18 months of age but his developmental milestones were otherwise normal. At 3 years of age he was investigated for restricted movement of his left arm and found to have a proximal radio-ulnar synostosis. Radiographs also demonstrated fusion of his C5/6 vertebral bodies. At 5 years of age he was investigated for an abnormal head shape and found to have craniosynostosis. His general health was otherwise normal and there had been no concerns about his developmental progress or behavior. On presentation to the craniofacial service at 6 years of age he was found to have pansynostosis with copper beating and mild blurring of the disc margins which remained unchanged over 4 months. His height, weight and head circumference were between the 50-75^th^ percentile. He had hypertelorism, exorbitism, malar hypoplasia and broad thumbs. He has not had surgery to date but continues to have monitoring for evidence of raised intracranial pressure. A renal ultrasound was normal. Molecular testing for craniosynostosis showed a heterozygous ERF c.1390_1391dupCC p.(K465Lfs*67) mutation.

**Patient 16 (Kindred 4)**

The father of the proband was noted to have hypertelorism, exorbitism and a sagittal ridge and was found to carry the same ERF mutation.

**Patient 17 (Kindred 5)**

**ERF c.248G>A p.(R83Q)**

This 7 year old girl is the first child of unrelated parents of Pakistani and white Caucasian origin. There were no concerns during pregnancy or the neonatal period and early developmental milestones were normal. She presented to the craniofacial unit at 3 years, 6 months with abnormal head shape, snoring and gross and fine motor delay. She was found to have ridged coronal and sagittal sutures and 3D CT scanning showed pansynostosis with cerebellar tonsillar descent. On examination, she had a head circumference of 52.6cm (75-91^st^ centile) with hypertelorism, exorbitism, malar hypoplasia and medial epicanthic folds. She had normal limbs other than mild clinodactyly of her 4^th^ toes. She wears glasses for hypermetropia. There are no concerns about her hearing. She is having speech and language therapy within her mainstream school. At 4 years of age she had adeno-tonsillectomy for sleep apnoea with symptomatic improvement. Molecular testing showed a heterozygous ERF c.248G>A p.(R83Q) missense change.

**Patient 18 (Kindred 5)**

The father of the proband was born at 39 weeks gestation by Caesarean section for cephalopelvic disproportion weighing 2.95kg. There were no neonatal concerns other than macrocephaly (head circumference on 98^th^ centile) for which he was followed by a pediatrician. He presented at 10 years of age with headaches and visual disturbance and was found to have bicoronal and sagittal synostosis with chronically raised ICP for which he had calvarial remodeling. He had recurrence of his headaches 12-18 months later and was found to have a Chiari malformation. On examination at 15 years of age he had a height on the 3^rd^ centile, weight on the 75-90^th^ centile. He had a broad forehead and face with orbital hypertelorism, down slanting palpebral fissures, medial epicanthic folds, exorbitism, malar hypoplasia and broad thumbs. He was suspected to have Crouzon syndrome but his molecular testing at that time was normal. He was subsequently found to carry the same ERF mutation as his daughter. He has no prior family history of craniosynostosis but his parents were not available for testing.

**Patient 19 (Kindred 6)**

**ERF c.1201_1202del; p.(K401Efs*10)**

This 7 year old boy was born to unrelated parents with no family history of note. The pregnancy was unplanned and there was a history of alcohol exposure during the first trimester. He was delivered by forceps at term weighing 3.125kg. He did not require resuscitation. He was noted to have a high palate and some hypotonia but fed well. He was referred for a pediatric evaluation at 7 months due to concerns about snuffly breathing and motor delay. His weight was on the 25^th^ centile with a head circumference on the 9-25^th^. He was noted to have skull asymmetry without palpable sutural ridging, a broad slightly bossed forehead, mild hypertelorism, a depressed nasal bridge, long philtrum, small mouth with a high narrow palate, low set posteriorly rotated ears, inverted nipples and mild 5^th^ finger clinodactyly. An MRI brain scan was reported as showing ventriculomegaly. He began walking unsupported at 18 months of age but had speech delay for which he has speech and language therapy. He presented to the craniofacial unit at 28 months of age due to concerns about his head shape and was found to have pansynostosis with raised intracranial pressure for which he has had a VP shunt. His vision and hearing were normal. On examination he had a long head with sutural ridging and visible blood vessels over the forehead. He had a flat facial profile with mild facial asymmetry, a sacral dimple, slightly broad great toes and an overriding second toe on the left.

**Patient 20 (Kindred 7)**

**ERF c.202G>C; p.(G68R)**

This is one of non-identical twin boys born to healthy unrelated Caucasian parents. The pregnancy was complicated by maternal pre-eclampsia and both twins were breech presentation. They were delivered by Caesarean section at 34 weeks gestation. This child was found to have macrocephaly with ventriculomegaly, dysplastic auricles and sagittal and right lambdoid synostosis for which he underwent calvarial remodeling at 6 months of age. Developmentally, he sat unsupported from 9 months of age and walked from 16 months. However, his speech was delayed at 19 months with only a few single words. Molecular genetic testing for Muenke syndrome, FGFR2 exons 8 and 10, and for Saethre-Chotzen syndrome as well as array-CGH chromosome testing did not identify any abnormality. Subsequent ERF testing showed an ERF c.202G>C; p.(G68R) missense mutation which had been paternally inherited.

**Patient 21 (Kindred 7)**This patient is the dizygotic twin brother of patient 20. He was assessed as having metopic synostosis and slightly dysplastic auricles, but has not had craniofacial surgery. Developmentally, like his twin, he sat unsupported from 9 months of age, walked from 16 months but had only a few single words at 19 months. He was found to have inherited the same ERF mutation as his twin.

**Patient 22 (Kindred 7)**

The father of patients 20 and 21 was investigated as a baby for an abnormal head shape but did not have any surgery. He is in good general health with no history of learning or behavioural problems. He had a rather narrow head and a head circumference of 57cm. He was found to carry the same ERF mutation as his twin sons.

**Patient 23 (Kindred 8)**

**ERF c.161A>G; p.(E54G)**

This boy is the only child of unrelated Caucasian parents. He was delivered by elective Caesarean section for placenta praevia at 36 weeks gestation. There were no significant neonatal problems other than progressive skull asymmetry. At 7 months of age when he presented to the craniofacial unit he had been in good general health. He had a head circumference of 44.7cm with marked skull asymmetry, a short upturned nose and medial epicanthic folds. He had otitis media with effusions but without significant hearing impairment, and a pseudo-squint. Developmental assessment at 8 months of age using Bailey scales of infant and toddler development (3^rd^ Ed) scored his abilities within the average range. A CT head scan confirmed a left lambdoidal craniosynostosis with mild ventriculomegaly of the lateral and third ventricles, and a reduced posterior fossa volume but no Chiari malformation. Posterior skull vault remodeling was undertaken at 8 months of age.

**Patient 24 (Kindred 8)**

The mother of patient 23 was found to have a mildly scaphocephalic head shape, with mild hypertelorism and exorbitism. Her mother and one of her 10 siblings were felt to have a similar facial appearance but none had a formal diagnosis of craniosynostosis. She was found to carry the same heterozygous ERF mutation as her son but other family members have not been tested to date.

**Patient 25 (Kindred 9)**

**ERF c.1201_1202delAA; p.(K401Efs*10)**

This girl is the first child of unrelated parents and has 4 healthy maternal half-siblings. The pregnancy was normal until 37 weeks gestation when she was delivered by elective Caesarean section because of maternal illness. There were concerns about her head shape from soon after birth and investigations found a right coronal craniosynostosis for which she had fronto-orbital advancement and remodeling at 13 months of age. Around 3 years of age she developed increasing headaches accompanied by vomiting. A CT scan showed low cerebellar tonsils but no Chiari malformation and the ventricles appeared normal in size. She was found to have raised intracranial pressure on direct monitoring for which she underwent calvarial remodeling at 3.5 years of age. Her motor and speech milestones were within normal but she had behavioural problems with temper control, hyperactivity and sensory processing problems in the autistic spectrum. At 38 months of age she had a head circumference of 50 cm. She had a short, upturned nose with a relatively long philtrum, broad halluces and mild joint hypermobility. Genetic testing including array-CGH chromosomes, FGFR1 exon 7, FGFR2 exons 8 and 10, FGFR3 exons 7 and 10 and TWIST sequencing and MLPA did not identify any abnormality but she was found to have the above heterozygous pathogenic ERF mutation.

**Patient 26 (Kindred 9)**

The father of patient 25 was noted to have hypertelorism, mild malar hypoplasia and mild relative prognathism but a normal looking head shape. He was subsequently found to share the same ERF mutation as his daughter.

**Patient 27 (Kindred 10)**

**ERF c.547C>T; p.(R183*)**

This is the first child of unrelated Caucasian parents. She was delivered normally at term after a normal pregnancy. There were no neonatal problems. She first presented to her paediatrician at 31 months of age with global developmental delay. Her array-CGH chromosome testing showed a 16p13.11 recurrent micro duplication which is a recognized neuro-susceptibility factor which may contribute to intellectual disability and behavioral abnormalities. Her biochemical screening, including complete blood count, electrolytes, liver function, thyroid function, CK, ferritin, plasma amino-acids and urine amino and organic acids and GAGs, were normal. She was found to have a myopia and astigmatism. Her hearing assessments were normal.

At 5 years, 6 months of age she was noted to have mild exorbitism and a Crouzonoid facial appearance and she was referred to the craniofacial service. She was microcephalic with a head circumference of 48.9cm (<2^nd^ centile). A CT head scan confirmed a non-scaphocephalic sagittal craniosynostosis. There has been no evidence of raised ICP on ICP monitoring and she has not had any operative intervention to date. She has significant speech delay, problems with social development and has been assessed as having moderate learning disability with possible autistic spectrum disorder for which she has additional support in mainstream school.

She was found to carry a heterozygous ERF c.547C>T; p.(R183*) mutation. Her father was noted to have mild exorbitism but her parents have not taken up genetic testing to date.

**Patient 28 (Kindred 11)**

**ERF c.547C>T; p.(R183*)**

This girl is the first of 3 children born to unrelated Caucasian parents. She also has a maternal half-sister. She was delivered normally after a normal pregnancy weighing 3.4kg and there were no neonatal problems. Her early developmental milestones were within normal limits but she experienced difficulties with fine motor tasks and was identified as having mild learning problems at school. Formal assessment found that she had reduced processing speeds, verbal memory and difficulties with verbal tasks. At 15 years of age she developed generalized seizures treated with Levetiracetam. She was noted to have a Crouzonoid facial appearance with exorbitism and malar hypoplasia. She was undergoing orthodontic treatment for a class III occlusal relationship and overcrowding. Her lateral metatarsals were short but her hands were normal. A CT scan showed sagittal and bilambdoidal craniosynostosis but the intracranial appearances were normal. Intracranial pressure monitoring was normal and she has not required any operative intervention.

Genetic investigation found that she had a heterozygous ERF c.547C>T; p.(R183*) mutation.

**Patient 29 (Kindred 11)**

The brother of patient 28 was tested as part of the family follow-up and found to carry the same ERF mutation. He was reported to have learning and behavioral problems and to wear hearing aids. He had a head circumference of 54.3cm and radiologically confirmed sagittal and bilambdoidal craniosynostosis. He had hypertelorism, exorbitism and low set ears. He had short lateral metatarsals but normal hands.

**Patient 30 (Kindred 11)**

The father of patients 28 and 29 gave a past medical history of adeno-tonsillectomy but was otherwise in good health. He had received no additional support in mainstream school but finished at 16 years without any formal qualifications. He was employed as a domestic cleaner. He had a normal head shape but a Crouzonoid facial appearance and was found to carry the same ERF mutation as his children. His mother was said to share a similar facial appearance but had no history of craniofacial investigations or surgery and she has not undergone testing to date.

**Patient 31 (Kindred 12)**

**ERF c.157G>A; p.(G53R)**

This is the only child of unrelated parents of Indian ethnicity. There is a strong maternal family history of Charcot-Marie-Tooth (CMT1B) with a confirmed MPZ mutation. The proband was delivered by emergency Caesarean section for fetal distress at 36 weeks gestation following a normal pregnancy weighing 4lb. His developmental milestones were all delayed. He first sat unsupported at 1 year and walked from 3 years. His first words were after 2 years of age. He presented at 4 years, 3 months of age with concerns about his developmental delay and suspected CMT1B. He was noted to have a height of 94 cm with a weight of 14.7kg and a head circumference of 54.8cm. He had a prominent forehead with frontal bossing, hypertelorism with down-slanting palpebral fissures, an open mouth appearance and low-set ears. His array-CGH chromosome testing was normal. A skull X-ray revealed sagittal craniosynostosis. He was found to have a heterozygous ERF c.157G>A; p.(G53R) variant.

The proband was noted to resemble his mother who has a history of mild learning difficulties and long-standing headache. She has not had genetic testing to date.

**Patient 32 (Kindred 13)**

**ERF c.652C>T; p.(R218*)**

This 4 year old boy is the first child of unrelated parents, both of whom have mild proportionate short stature. The pregnancy was normal. He was delivered normally at 38 weeks gestation following ruptured membranes 31 hours previously. His birth weight was 2.7kg and head circumference 32cm. He developed respiratory distress soon after birth and evidence of a congenital pneumonia for which he was treated with intravenous antibiotics and required ventilation for 2 days. Cranial ultrasound was normal and echocardiogram showed only a small patent ductus arteriosus. He was discharged home at 10 days of age, feeding normally and passed his new-born hearing screen.

He re-presented at 10 months of age with plagiocephaly, poor weight gain and globally delayed milestones. At that time his weight and length were tracking along the 0.4^th^ centile while his head circumference measurements fell between the 50-75^th^ centiles. He was noted to have a large anterior fontanelle, hypertelorism, limb hypotonia but normal mid-face, clavicles, axial tone, power and reflexes. Investigations which included a-CGH chromosome testing, CK, plasma lactate, plasma and urine amino-acids, urine organic acids, very long chain fatty acids, plasma sterols, plasma and lysosomal enzyme screening, transferrin isoelectric focusing, blood spot carnitines, thyroid function, growth hormone and 25-hydroxy vitamin D were normal and he was not felt to have a primary neuromuscular or metabolic disorder. A skull X-ray at 16 months of age showed a widely patent anterior fontanelle and no radiological evidence of craniosynostosis. His plagiocephaly was assessed as positional. He was noted to have very blonde hair and skin for his family and ophthalmology investigations revealed foveal hypoplasia with slightly reduced central acuity and prominent crossed asymmetry on electro-diagnostic testing indicating chiasmal misrouting and consistent with mild oculo-cutaneous albinism.

He was re-assessed at 26 months of age when he had been walking independently since 19 months. He was using about 10 recognizable words and would follow simple commands. A skeletal survey showed delayed bone age (estimated 9 months at a chronological age of 26 months; standard deviation 4 months), but no other features to suggest a skeletal dysplasia.

On re-assessment at about 2 ½ years of age he had a height of 87.5cm (2-9^th^ centile) with a head circumference of 51.2cm (50^th^ centile). His fontanelle had closed. He had hypertelorism and mild exorbitism. Genetic testing at that stage excluded Muenke syndrome and any detectable mutations in FGFR2 on extended screen but he was found to have a maternally inherited heterozygous nonsense ERF mutation, ERF c.652C>T; p.(R218*) which has not been reported previously but is predicted to be pathogenic.

When last assessed at 4 years of age, he had been in good general health other than longstanding constipation treated with Movicol on alternate days. He had significantly delayed language skills despite speech and language therapy. Assessment using a preschool language scale 4 UK (PLS-4UK) gave a receptive language score of 65 (normal range 85-115). He was not co-operative with expressive language testing but had a large vocabulary and was making 2-3 word phrases. He was sociable but had persistent problems with poor attention and concentration. He attends mainstream school with 1:1 support. Although unable to fully co-operate, no significant hearing or visual impairment has been found. Head CT scan at 39 months has shown sagittal, bilambdoidal and fronto-sphenoidal craniosynostosis with mild plagiocephaly but normal intracranial appearances. He has normal class 1 occlusion. Ophthalmic monitoring has not shown any evidence of raised intracranial pressure to date.

**Patient 33 (Kindred 13)**

The mother of the proband has a history of delayed speech and mild learning difficulties. She has a normal head shape but mild hypertelorism. She was found to carry the same ERF mutation. She has no preceding family history of craniosynostosis but her parents have declined genetic testing.

**Patient 34 (Kindred 14)**

**ERF c.247C>T; p.(R83W)**

This is a 9 year old boy who was referred to our craniofacial service at 8 years 5 months of age with a pathogenic heterozygous ERF mutation identified as part of a research exome sequencing project. He had been recruited because of delayed speech and language milestones, learning difficulties and dysmorphism. He had been fostered from 6 weeks of age and subsequently adopted. His biological father was known to have had a hearing impairment. He was delivered by Caesarean section at 31 weeks gestation and remained in hospital until 6 weeks of age. There were no concerns about his head shape at birth. He suffered from gastro-oesophageal reflux treated medically with thickened feeds and omeprazole until around 5 years of age, but weaned normally and eats a normal diet. He failed his new-born hearing test and subsequent audiology investigations have shown that he has bilateral sensorineural hearing loss, particularly affecting the mid-frequencies, for which he wears hearing aids. He has microscopic haematuria and proteinuria with a normal renal ultrasound which are being investigated separately.

He walked at 18-19 months of age. His speech was delayed but improved with speech and language therapy. At 9 years of age he speaks clearly and in full sentences. He attends mainstream school where he has support from a hearing impairment advisor and teaching assistant, and small group teaching. His learning difficulties are felt to be mild. There have been no significant problems with his concentration or behavior.

On examination at 9 years of age he had a height of 131.4cm (25-50^th^ centile) and a head circumference of 55.4cm (>97^th^ centile). He had a mildly scaphocephalic head shape with a bony convexity overlying the closed anterior fontanelle. He had hypertelorism with exorbitism but his mid-face and occlusion were normal. The examination was otherwise normal.

His cranial CT scan on presentation showed sagittal and bilambdoidal craniosynostosis. His ophthalmology assessments have not shown evidence of raised intracranial pressure to date.

The research findings were confirmed in our diagnostic laboratory. He has a heterozygous ERF c.247C>T; p.(R83W) mutation which has been shown to be maternally inherited although no clinical information is available about his mother or biological family on that side. He also has bi-allelic sequence variants in MYO15A one of which is a known pathogenic mutation predicted to be protein truncating while the other is a predicted missense variant of uncertain significance. It is not clear currently whether these variants explain his sensorineural hearing impairment but his hearing loss is suspected to be coincidental to his ERF-related craniosynostosis.

**Patient 35 (Kindred 15)**

**ERF N/c.1270C>T; p.(Q424*)**

This 15 month old girl is the second child of unrelated parents with no significant family history. She was noted to have scaphocephaly at 3 weeks of age when she presented to hospital with varicella zoster. Investigations confirmed that she had a sagittal and bilambdoidal craniosynostosis for which she had a posterior skull vault expansion aged 8 months. She suffered from gastro-oesophageal reflux which was treated medically and has resolved. She has had frequent upper respiratory infections and evidence of otitis media with effusions at 13 months. Otherwise, she has mild hypertelorism. Her general health and developmental progress have been normal. Neither parent has any clinical evidence of craniosynostosis or facial resemblance to the proband but the samples from the parents for testing are awaited.

**Patient 36 (Kindred 16)**

**c.1A>T; p.?**

This 14 year old boy is the youngest of 4 children. He presented at 24 months of age with facial dysmorphism (orbital hypertelorism, mild exorbitism, malar hypoplasia) and broad thumbs and 1^st^ toes. He exhibited papilledema and raised ICP. Imaging revealed a pansynostosis. He underwent posterior vault expansion at 28 months. Subsequent follow up has revealed mild expressive, receptive and speech delay but no motor or behavioral concerns. His 3 elder siblings are clinically unaffected. The parents have not volunteered for testing.
